# Clinical and chest computed tomography features associated with severe *Chlamydia psittaci* pneumonia diagnosed by metagenomic next-generation sequencing: A multicenter, retrospective, observational study

**DOI:** 10.1097/MD.0000000000032117

**Published:** 2022-12-16

**Authors:** Limin Xu, Ziwen Zhao, Hui Mai, Xiaoying Tan, Yubin Du, Changquan Fang

**Affiliations:** a Department of Geriatrics, Huizhou First People’s Hospital, Huizhou, People’s Republic of China; b Department of Pulmonary and Critical Care Medicine, Guangzhou First People’s Hospital Affiliated to South China University of Technology, Guangzhou, People’s Republic of China; c Department of Pulmonary and Critical Care Medicine, Huizhou Central People’s Hospital, Huizhou, People’s Republic of China.

**Keywords:** *Chlamydia psittaci*, metagenomic next-generation sequencing, pneumonia, psittacosis, severity

## Abstract

*Chlamydia psittaci* pneumonia is a rare disease with varying clinical presentations. Here, we aimed to investigate the clinical and chest computed tomography (CT) features of severe psittacosis pneumonia. Clinical data of 35 patients diagnosed with psittacosis pneumonia were retrospectively analyzed using metagenomic next-generation sequencing. The patients were classified into severe (n = 20) and non-severe (n = 15) groups. The median age of patients was 54 years, and 27 patients (77.1%) had a definite history of bird contact. Severe patients had more underlying comorbidities and were more prone to dyspnea and consciousness disorders than non-severe patients. The neutrophil count and D-dimer, lactate dehydrogenase, interleukin (IL)-2, IL-6, and IL-10 levels were higher, whereas the lymphocyte, CD3^ + ^T cell, and CD4^ + ^T cell counts, CD4^+^/CD8^ + ^T cell ratio, and albumin level were substantially lower in severe patients than in non-severe patients. Chest CT findings of severe patients revealed large areas of pulmonary consolidation, and ground-glass opacities were observed in some patients, with a higher risk of involving multiple lobes of the lungs and pleural effusion. One patient died of multiple organ failure, whereas the condition of the other 34 patients improved, and they were discharged from the hospital. Patients with severe psittacosis pneumonia often have underlying comorbidities and are prone to developing dyspnea, consciousness disorder, and lesions in both lungs. Serum D-dimer, IL-2, IL-6, and IL-10 levels and lymphocyte, CD3^ + ^T cell, and CD4^ + ^T cell counts are associated with disease severity.

## 1. Introduction

*Chlamydia psittaci* belongs to the genus *Chlamydia*, which comprises gram-negative obligate intracellular bacteria including *Chlamydia pneumoniae* and *Chlamydia trachomatis*. *C psittaci* infection often results in severe pneumonia and even multiple organ failure owing to its strong pathogenicity.^[1]^ Pneumonia caused by *C psittaci* is a rare disease, accounting for approximately 1% of community-acquired pneumonia cases.^[2]^ The number of reported cases is relatively small, mainly because of the limitations of etiological assessment techniques and insufficient clinical information on the disease.^[1,3]^

Unlike the conventional methods used for etiological assessment, including isolation and culture of pathogenic microorganisms, serological testing, and polymerase chain reaction, metagenomic next-generation sequencing (mNGS) can be used for direct high-throughput sequencing of nucleic acids in clinical samples. It can help identify unknown pathogens, pathogens that are difficult to culture, such as *C psittaci*, and fungi and pathogens causing mixed infections.^[4]^

In recent years, clinical reports of psittacosis pneumonia diagnosed using mNGS have increased.^[5]^ However, the number of reported cases is small, most of which have been descriptive,^[5]^ and a few systematic analyses of the clinical features of severe and non-severe patients have been conducted. The aim of this study was to investigate the clinical and chest computed tomography (CT) features of severe psittacosis pneumonia. For this purpose, we retrospectively analyzed the clinical data of 35 patients with psittacosis pneumonia admitted to 4 tertiary A hospitals in South China between January 2020 and April 2022 and compared the clinical features of severe and non-severe patients.

## 2. Methods

### 2.1. Study design and participants

In this study, we retrospectively analyzed 35 patients diagnosed with psittacosis pneumonia using mNGS between January 2020 and April 2022 and admitted to 4 tertiary A hospitals in South China. The patients were classified into the following 2 groups based on the severity of pneumonia: severe (n = 20) and non-severe (n = 15) groups. The diagnostic and clinical classification of psittacosis pneumonia was based on the Consensus Guidelines on the Management of Community-Acquired Pneumonia in Adults, developed by the Infectious Diseases Society of America and the American Thoracic Society in 2007; severe pneumonia was defined as the presence of either one major criterion or 3 or more minor criteria.^[6]^ *C psittaci* DNA was detected in the alveolar lavage fluid or venous blood of all patients using mNGS, and conventional etiological assessments including blood, sputum, and alveolar lavage fluid smears and cultures did not detect other pathogens. Patients aged <18 years, who were pregnant, with thrombotic diseases or HIV infection, and/or with severe data loss, were excluded.

### 2.2. Ethics

This study was approved by the Ethics Review Committee of Huizhou First People’s Hospital and was conducted in accordance with the provisions of the Declaration of Helsinki (Approval 2020107). All patients and legally authorized representatives or next of kin of the deceased patients provided informed consent for this study.

### 2.3. Data collection

General information of all patients, including sex, age, underlying diseases, and epidemiological history, was collected. The clinical symptoms and signs of the patients, blood test results, chest CT findings, mNGS results, treatment, and outcome (survival or death) were recorded. The modified British Thoracic Society’s pneumonia score (CURB-65),^[7]^ pneumonia severity index (PSI),^[8]^ and acute physiology and chronic health evaluation II^[9]^ score upon hospital admission were calculated.

### 2.4. Detection of pathogens using metagenomic next-generation sequencing

The alveolar lavage fluid and venous blood samples were collected in strict accordance with standard clinical operation procedures, and the Ion AmpliSeq Kit (Guangzhou DARUI Biotechnology Co., Ltd., Guangzhou, China) was used for DNA extraction. DNA was randomly cut into 200 to 300-bp fragments. The insert size and adapter sequence were controlled to form a single-stranded ring structure via ligation using the Daan Universal DNA Library Construction Kit (Guangzhou DARUI Biotechnology Co., Ltd.). The prepared DNA nanospheres were loaded onto the sequencing chip and subjected to sequencing and bioinformatic analysis using the DA8600 high-throughput sequencer (Daan Gene Co., Ltd., Guangzhou, China).

### 2.5. Statistical analysis

All statistical analyses were performed using SPSS version 19.0 (IBM Corp, Armonk, NY). Quantitative variables are described as mean (standard deviation) or median (quartiles). A *t* test was used for intergroup comparisons if the variables were normally distributed, and Mann–Whitney *U* test was used if the variables were not normally distributed. Categorical variables are described as frequency rate and percentage, and the *χ*^2^ test or Fisher’s exact test was used for comparisons between groups. Statistical significance was set at *P* < .05.

## 3. Results

### 3.1. Demographic and clinical characteristics

Of the 35 patients, 16 were males and 19 were females. The median age was 54 years with a range of 30 to 88 years, and 27 patients (77.1%) had a definite history of bird contact. Disease onset was mostly in winter (57.1%, 20 cases). The severe group had higher CURB-65, PSI, and acute physiology and chronic health evaluation II scores and more underlying diseases, and were more prone to dyspnea and consciousness disorders than did the non-severe group (Table 1, *P* < .05).

**Table 1:** Baseline characteristics of patients with *Chlamydia psittaci* pneumonia (severe and non-severe groups).

Characteristic	Severe	Non-severe	*P*
	(n = 20)	(n = 15)	
**Demographics**			
Age, years, mean ± SD	58.7 ± 15.4	56.2 ± 7.6	.608
Female, n (%)	9 (45.0)	10 (66.7)	.203
History of exposure to avian, n (%)	16 (80.0)	11 (73.3)	.642
Illness onset to diagnosis, days, mean ± SD	10.0 ± 3.9	6.5 ± 2.6	.063
Illness onset to Chest CT, days, mean ± SD	6.5 ± 2.7	3.7 ± 2.4	.057
Admission to diagnosis, days, mean ± SD	3.7 ± 2.4	2.7 ± 0.7	.316
CURB-65, points, mean ± SD	2.6 ± 1.1	0.4 ± 0.8	<.001
PSI, points, mean ± SD	126.7 ± 28.8	67.8 ± 14.2	<.001
APACHE Ⅱ, points, mean ± SD	17.4 ± 6.2	6.7 ± 2.3	<.001
PO_2_/FiO_2_ ration, mm Hg, mean ± SD	191 ± 40.8	390.8 ± 28.0	<.001
**Underlying disease, n (%**)	13 (65.0)	4 (26.7)	.025
Hypertension	5 (25.0)	2 (13.3)	.393
Diabetes	6 (30.0)	1 (6.7)	.088
Coronary heart disease	3 (15.0)	2 (13.3)	.889
COPD	2 (10.0)	1 (6.7)	.727
**Initial signs and symptoms, n (%**)			
Fever > 39.0 ℃	19 (95.0)	13 (86.7)	.383
Cough, hypodynamia, anorexia	20 (100.0)	14 (93.3)	.241
Dyspnea	20 (100.0)	5 (33.3)	<.001
Headache, Myalgia	11 (55.0)	8 (53.3)	.922
Disorder of consciousness	5 (25.0)	0	.036
**Season of admission, n (%**)			
Spring	2 (10.0)	1 (6.7)	1.0
Summer	2 (10.0)	3 (20.0)	.631
Autumn	3 (15.0)	4 (26.7)	.430
Winter	13 (65.0)	7 (46.6)	.321

Data are presented as mean ± SD, median (quartiles), or no. (%). No. refers to the number of patients with available data.

APACHE = acute physiology and chronic health evaluation, COPD = chronic obstructive pulmonary disease, CT = computed tomography, CURB-65 = British Thoracic Society’s modified pneumonia score, SD = standard deviation, PSI = pneumonia severity index.

We detected *C psittaci* DNA in the alveolar lavage fluid (30 cases) and venous blood (5 cases) samples of patients using mNGS. Among them, 11 patients presented this pathogen alone, and the pathogen was more common in non-severe cases. Other pathogenic microorganisms such as *Staphylococcus aureus*, *Pseudomonas aeruginosa*, *Acinetobacter baumannii*, *Klebsiella pneumoniae*, *Candida albicans*, and Epstein–Barr virus were also detected in the remaining 24 patients, and were more common in severe cases. The number of reads of *C psittaci* ranged from 3 to 14 940.

### 3.2. Laboratory and radiological data

The neutrophil count and D-dimer, lactate dehydrogenase, interleukin (IL)-2, IL-6, and IL-10 levels were significantly higher (*P* < .05), whereas the lymphocyte, CD3^ + ^T cell, and CD4^ + ^T cell counts, CD4^+^/CD8^ + ^T cell ratio, and albumin level were significantly lower (*P* < .05) in the severe group than in the non-severe group (Table 2).

**Table 2:** Laboratory findings of patients with *Chlamydia psittaci* pneumonia (severe and non-severe groups).

Laboratory variable	Normal range	Median (Quartiles)		*P*
		Severe (n = 20)	Non-severe (n = 15)	
Leukocyte, ×10^9^/L	4.0–10.0	9.4 (6.6–14.1)	9.0 (8.2–10.8)	.314
Neutrophil, %	45.0–75.0	90.5 (86.0–93.6)	73.9 (63.6–83.3)	.020
Lymphocyte count, ×10^9^/L	1.1–3.2	0.47 (0.30–0.55)	1.17 (0.93–1.50)	<.001
C-reactive protein, mg/L	0–5	195.5 (93.3–306.2)	129.5 (97.0–244.0)	.316
Procalcitonin, ng/mL	0–0.5	0.93 (0.50–14.16)	0.29 (0.17–0.58)	.057
D-dimer, ng/mL	0–500	7540 (4540–12015)	1233 (582–1852)	<.001
Lactate dehydrogenase, U/L	109–245	425 (289–578)	243 (194–433)	.042
Creatine kinase, U/L	40–200	360 (99–1317)	61 (29–143)	.147
Alanine aminotransferase,U/L	0–40	46.5 (36.0–63.2)	36.0 (18.5–108.7)	.701
Albumin, g/L	40–55	29.5 (23.8–33.5)	34.7 (32.1–38.7)	.020
Serum urea nitrogen, mmol/L	2.9–8.2	5.4 (3.6–8.5)	4.2 (3.5–6.0)	.222
Serum creatinine, µmol/L	62–106	68.5 (52.0–90.2)	66.5 (51.5–77.0)	.389
**Lymphocyte subpopulation**				
CD3^ + ^count,/µL	770–2860	404 (354–670)	1028 (848–1328)	.016
CD4^ + ^count,/µL	500–1440	220 (174–318)	648 (532–796)	.008
CD8^ + ^count,/µL	238–1250	164 (138–366)	296 (168–444)	.142
CD4/CD8	1.0–2.47	1.38 (0.96–1.75)	2.05 (1.77–3.18)	.016
**Cytokines**				
Interleukin-2, pg/mL	0–5.71	1.02 (0.38–1.39)	0.42 (0.15–0.79)	.045
Interleukin-4, pg/mL	0–2.80	1.27 (0.41–2.01)	0.65 (0.01–1.55)	.273
Interleukin-6, pg/mL	0–5.30	368 (197–1000)	33.4 (18.2–121.3)	<.001
Interleukin-10, pg/mL	0–4.91	4.83 (8.93–12.77)	2.60 (2.31–3.54)	.002
Tumor necrosis factor-α, pg/mL	0–4.60	1.05 (0.78–1.90)	0.65 (0.23–1.68)	.193
Interferon-γ, pg/mL	0–7.42	4.42 (0.26–41.32)	1.40 (0.85–5.49)	.457

Data are presented as median (quartiles).

**Figure 1.**
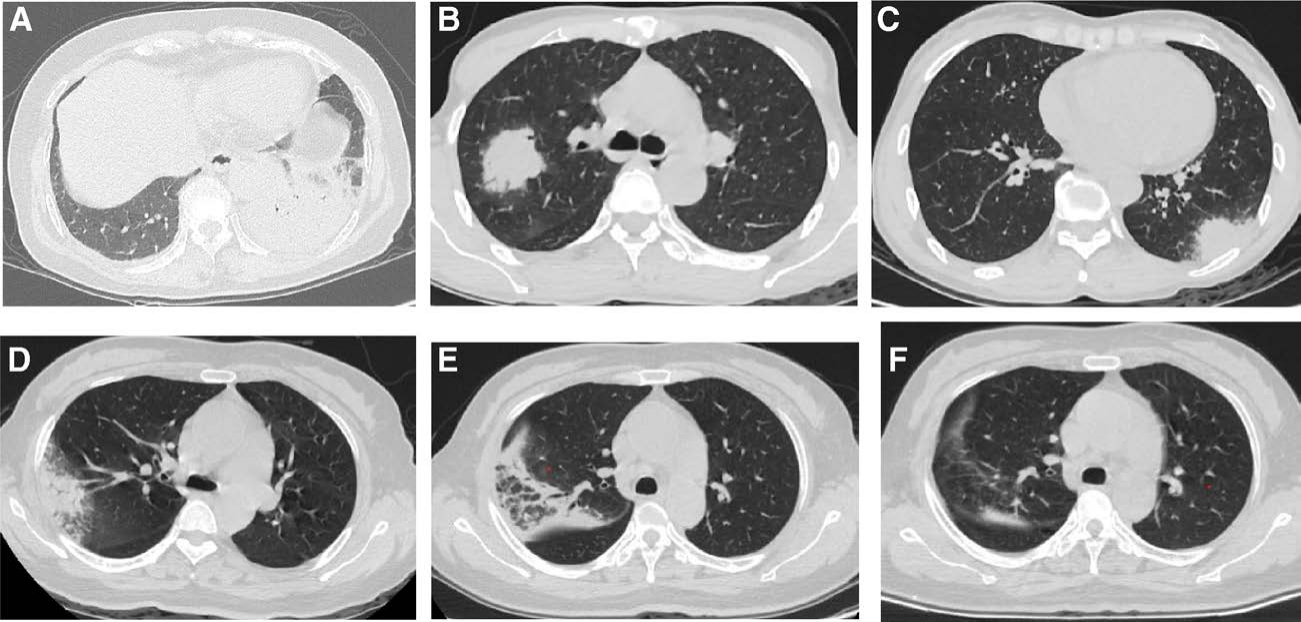
Chest computed tomography (CT) findings of non-severe *Chlamydia psittaci* pneumonia. (A) A 67-year-old female presenting a fever for 3 days, large air-space consolidation in the left lower lobe. (B) A 51-year-old male presenting a fever for 3 days, focal consolidation in the right upper lobe. (C) A 48-year-old female presenting a fever for 2 days, focal consolidation in the left lower lobe subpleural area. (D–F) A 51-year-old female with the initial CT scan performed 3 days after onset showed focal consolidation in the right upper lobe subpleural area (D). The lesions increased, and the scope expanded following treatment with levofloxacin for 7 days (E). Thereafter, the lesion evidently shrank and became less dense following treatment with moxifloxacin for 7 days (F).

**Figure 2.**
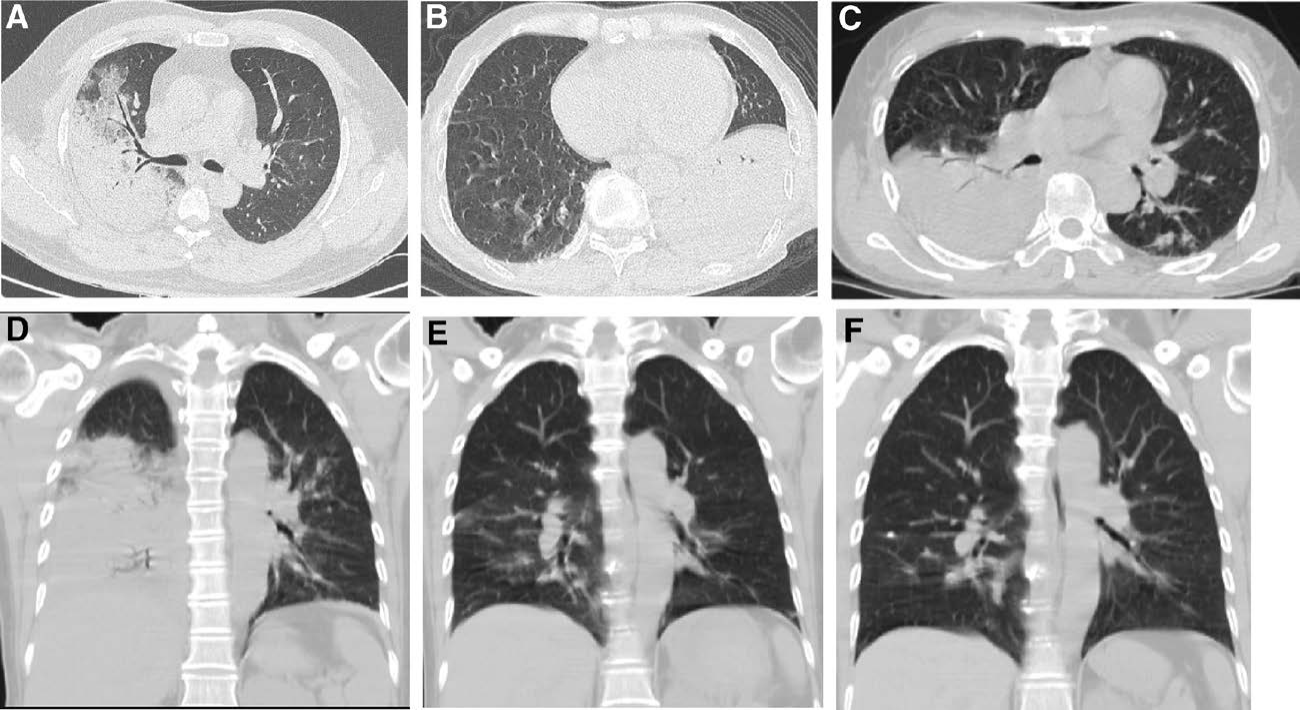
Chest computed tomography (CT) findings of severe *Chlamydia psittaci* pneumonia. (A) A 52-year-old male presenting a fever for 3 days, large air-space consolidation and air bronchogram in the right upper lobe. (B) A 47-year-old male presenting a fever for 5 days, large air-space consolidation in the left lower lobe. (C) A 48-year-old male presenting a fever for 4 days, large air-space consolidation and multi-lobe ground-glass opacity in the bilateral lung. (D–F) A 58-year-old female with the initial CT scan performed 4 days after onset showed large air-space consolidation in the right lung (D). The lesion evidently shrank and became dense following treatment with moxifloxacin and doxycycline for 7 days (E). A follow-up CT scan performed 18 days after the onset did not show any consolidation (F).

All patients underwent chest CT after admission (Table 3), and the median time from the onset of the disease to the first chest CT was 5 days. The chest CT showed more frequent and severe exudation in the lower lobes. Consolidation was observed in all patients, and some patients presented ground-glass opacities and a small amount of pleural effusion, pericardial effusion, and mediastinal lymphadenopathy. The lesions in non-severe patients were generally confined to a single lung lobe (Fig. 1). The lesions often progressed rapidly in severe patients, resulting in a massive consolidation of multiple lobes in the lungs and pleural effusion (Fig. 2). After effective treatment, the lesions could be completely absorbed, or only a small amount of strip shadows remained (Figs. 1 and 2).

**Table 3:** Chest computed tomography manifestations of patients with *Chlamydia psittaci* pneumonia (severe and non-severe groups).

Variable	No. (%)		*P*
	Severe (n = 20)	Non-severe (n = 15)	
**Pattern**			
Consolidation	16 (80.0)	15 (100.0)	.066
Consolidation + Ground-glass opacity	4 (20.0)	0	.066
**Distribution**			
Unilateral, left lung	4 (20.0)	5 (33.3)	.372
Unilateral, right lung	2 (10.0)	5 (33.3)	.088
Bilateral	14 (70.0)	5 (33.3)	.031
Single lung lobe	0	10 (66.7)	<.001
Multiple lung lobe	20 (100.0)	5 (33.3)	<.001
Upper lobe	13 (65.0)	7 (46.7)	.278
Right middle lobe	7 (35.0)	2 (13.3)	.147
Lower lobe	20 (100.0)	11 (73.3)	.014
Lesions were mainly in the upper lobe	6 (30.0)	5 (33.3)	.833
Lesions were mainly in the lower lobe	14 (70.0)	10 (66.7)	.833
**Complications**			
Pleural effusion	15 (75.0)	4 (26.7)	.005
Pericardial effusion	3 (15.0)	0	.117
Lymph node enlargement	7 (35.0)	3 (20.0)	.331

Data are presented as No. (%). No. is the number of patients with available data.

### 3.3. Treatment and prognosis

All patients underwent mNGS examination after admission, and the median time from admission to diagnosis was 3 days (Table 1). Twenty-seven patients required respiratory support: 10 patients (3 severe and 7 non-severe patients) received oxygen through nasal catheters, 7 severe patients received high-flow nasal oxygen therapy, and 10 severe patients required mechanical ventilation with 4 patients receiving tracheal intubation and ventilation and 6 receiving noninvasive ventilation. All patients were treated with antibiotics before diagnosis. The common regimen was β-lactam/β-lactamase inhibitor combinations or carbapenem combined with quinolone. Antiviral drugs, including oseltamivir and arbidol, were administered to 13 patients, and 8 patients were treated with glycopeptide antibiotics. Of the 15 non-severe patients, 10 were initially treated with respiratory quinolones. After the detection of *C psitta*ci DNA using mNGS, 6 patients continued to use levofloxacin/moxifloxacin, 2 replaced levofloxacin with moxifloxacin owing to a poor response to treatment, and the remaining 7 were treated with doxycycline alone or in combination with other antibiotics. Twenty severe patients were initially treated with respiratory quinolones, of which only 6 showed a good response to treatment, and azithromycin was administered to one of the remaining patients. Tetracyclines were administered alone or with other antibiotics to 13 patients. Except for 1 patient who died of multiple organ failure, all patients showed improvements (Table 4), with their body temperatures gradually decreasing to normal levels in approximately 2 to 3 days.

**Table 4:** Treatment and outcomes of patients with *Chlamydia psittaci* pneumonia (severe and non-severe groups).

Variable	Severe (n = 20)	Non-severe (n = 15)	*P*
**Empirical antibiotics covered, n (%**)			
Quinolones	20 (100.0)	10 (66.7)	.005
**Effective antimicrobial therapy, n (%**)			
Quinolones	6 (30.0)	8 (53.3)	.163
Macrolides	1 (5)	0 (0)	1.0
Tetracycline	4 (20.0)	3 (20.0)	1.0
Tetracycline + Quinolones	9 (45.0)	4 (26.7)	.267
Duration of targeted anti-infective therapy, days, mean ± SD	16.0 ± 4.5	14.4 ± 2.5	.248
**Respiratory support, n (%**)	20 (100.0)	7 (46.7)	<.001
Oxygen therapy	3 (15.0)	7 (46.7)	.040
HFNC	7 (35.0)	0 (0)	.010
Noninvasive ventilation	6 (30.0)	0 (0)	.020
Invasive ventilation	4 (20.0)	0 (0)	.066
Sepsis, n (%)	15 (75.0)	0 (0)	<.001
ARDS, n (%)	7 (35.0)	0 (0)	.01
The length of hospital stay, days, mean ± SD	13.1 ± 6.2	8.8 ± 2.5	.123
Death, n (%)	1 (5.0)	0 (0)	1.0

Data are presented as No. (%). No. is the number of patients with available data.

ARDS = acute respiratory distress syndrome, HFNC = high-flow nasal cannula oxygen therapy, SD = standard deviation.

## 4. Discussion

Psittacosis pneumonia is a zoonotic disease caused by *C psittaci.* Humans generally lack immunity against the pathogen, and birds are the predominant hosts. Outbreaks caused by person-to-person transmission have been reported, but they are extremely rare. *C psittaci* is transmitted after the inhalation of aerosolized bacterial cells,^[10,11]^ which can rapidly cause severe respiratory failure and even death.^[5,12]^ High-risk factors for infection include old age, smoking, male sex, glucocorticoid use, and long-term residence in nursing homes. The prognosis of patients is directly associated with immunity. Timely and accurate diagnosis and treatment generally lead to a good prognosis.^[13,14]^ The present study showed that most patients had a history of bird contact, and the infection was more common in women and middle-aged people. Most patients had severe pneumonia and often had underlying comorbidities. However, the tracheal intubation rate and mortality rate recorded here were substantially lower than those reported by Tulzo.^[15]^ The possible reasons could be early diagnosis using mNGS, appropriate treatment, and the initial empirical use of quinolones, which improved the condition of some patients. In the past, the CURB-65 and PSI scores were mainly used to evaluate bacterial pneumonia.^[16]^ This study showed that the CURB-65 and PSI scores of patients with severe psittacosis pneumonia were significantly higher than those of non-severe patients, suggesting that the above-mentioned scores performed well in the evaluation of chlamydial pneumonia. *C psittaci* is a rare pathogen and an intracellular bacterium. When its DNA is detected in a sample using mNGS, the sample is considered positive for the bacterium.^[17]^ In this study, the effect of conventional anti-infection treatment in patients was not good before diagnosis. However, most patients had a good prognosis after the diagnosis of *C psittaci* infection was established and specific anti-infection treatment was administered.

Psittacosis is essentially a systemic infection. After the inhalation of the pathogen through the respiratory tract, it first proliferates in the reticuloendothelial system of the liver and spleen, and then enters the body through blood. Generally, the lungs are primarily affected, and the liver, kidney, and central nervous system could also be affected.^[1,18,19]^ Therefore, the clinical symptoms are diverse with varying severity levels. Common symptoms occurring in the patients evaluated in this study include high fever, cough, dyspnea, fatigue, anorexia, consciousness disorders, headache, and myalgia. Laboratory examinations revealed decreased lymphocyte counts and increased D-dimer, transaminase, lactate dehydrogenase, and creatine kinase levels, indicating that besides the respiratory system disease, the digestive system, neuromuscular system, hematological system, and coagulation function were affected to certain degrees. The more severe the disease, the more acute the organ dysfunction, and this is consistent with the reports of Chen et al^[20]^ and Su et al^[21]^ However, similar to the study of Branley et al,^[22]^ in the present study, only a small number of patients developed renal insufficiency, which might be owing to the small number of patients and timely and accurate diagnosis and treatment that prevented the occurrence and development of a cytokine storm.

Previous studies have paid little attention to the changes in coagulation function and inflammatory cytokines in psittacosis. In this study, the levels of D-dimer, IL-2, IL-6, and IL-10 in severe patients were remarkably higher, whereas the counts of CD3^ + ^T cells, CD4^ + ^T cells, and lymphocytes were substantially lower than those in non-severe patients. Increased levels of D-dimer indicate a hypercoagulable state and the possibility of vascular endothelial cell impairment.^[23]^ IL-10 is an anti-inflammatory cytokine, whereas IL-2 and IL-6 are pro-inflammatory cytokines. IL-6, which plays a critical regulatory role in the inflammatory response, is a key inflammatory mediator.^[24]^ Currently, the mechanism of a severe *Chlamydia* infection is not completely clear. Studies have shown that after the activation of the innate immune system by *Chlamydia* infection, it can promote the release of numerous pro-inflammatory mediators such as IL-6, IL-8, and tumor necrotic factor-α, inhibiting *Chlamydia* replication and accelerating pathogen elimination. The release of a large amount of pro-inflammatory factors can cause an excessive inflammatory response and coagulation dysfunction in patients. On the contrary, it can activate Treg, Th2, and other cells to secrete IL-10, IL-4, and other immunosuppressive cytokines, leading to a gradual increase in the anti-inflammatory response. A persistent inflammatory response can lead to increased lymphocyte apoptosis and immunosuppression.^[24–28]^ In summary, we believe that severe psittacosis pneumonia induces an excessive inflammatory response and immunosuppression in the early stage, and the lymphocyte counts and D-dimer, IL-2, IL-6, and IL-10 levels can help identify the severity of the disease.

The primary pathological basis for the chest radiologic features of psittacosis pneumonia is inflammatory cell infiltration in the alveolus with fibrinous exudate.^[29]^ The chest X-ray showed different degrees of exudation and consolidation, mainly involving the lower lobes of the lungs, and a small amount of pleural effusion can be observed in some patients.^[22,30]^ Most of the cases reported by Branley et al^[22]^ and Wen et al^[29]^ had single lung lesions, mostly located in the right lung. The chest CT findings of 48 patients reported by Shen et al^[31]^ mostly presented consolidation in a single lung and a single lobe, with a small amount of pleural effusion in a few patients. None of the above studies had analyzed the chest radiologic features of mild and severe patients separately. The chest radiologic findings of the patients in this study were mainly characterized using the degree of lung consolidation. The lesions of non-severe patients were generally confined to a single lung lobe, whereas severe patients mostly had consolidations in multiple lobes of both lungs with a small amount of pleural effusion, suggesting that the degree of consolidation and pleural effusion were closely related to the severity of the disease.

The first-choice treatment for psittacosis pneumonia is doxycycline, and other effective drugs include minocycline, azithromycin, moxifloxacin, and levofloxacin.^[11,13]^ In vitro experiments have confirmed the strong antimicrobial activity of the abovementioned drugs against *Chlamydia*.^[32,33]^ However, relevant randomized controlled clinical studies are still lacking. Previous studies on psittacosis pneumonia have reported inconsistent results owing to the small number of patients and lack of separate analysis of drug treatment for mild and severe patients.^[5,15,29,31]^ In this study, most patients received quinolones before diagnosis, of which some non-severe patients showed a good response to treatment, whereas severe patients showed a poor response. Therefore, using quinolones alone is not recommended in severe patients. Most patients received tetracyclines alone or in combination with other antibiotics and showed considerable improvement after diagnosis. Chen et al^[20]^ and Wu et al^[34]^ reported good efficacies of tetracyclines against severe psittacosis pneumonia. Therefore, tetracyclines should be the first choice for the treatment of psittacosis. If there are contraindications such as allergy, pregnancy, or use in children, macrolides can be chosen as an alternative treatment.^[22]^ However, considering the high-level resistance of *Chlamydia* to macrolides,^[35,36]^ only 1 patient was treated with macrolides in this study, and their clinical efficacy needs further verification.

A limitation of the study is that it was a retrospective study with a relatively small number of patients. As the conditions for polymerase chain reaction and serological detection of *C psittaci* were unavailable, all cases were confirmed using mNGS. Therefore, an in-depth, prospective study with a large number of patients is needed in the future.

## 5. Conclusion

In conclusion, patients with psittacosis pneumonia usually have a history of bird contact, and the common symptoms include high fever, cough, anorexia, fatigue, headache, and myalgia. Underlying comorbidities are common in severe patients, who are more prone to dyspnea, consciousness disorders, and multi-lobe lesions in both lungs than non-severe patients. The levels of serum D-dimer, IL-2, IL-6, and IL-10 and the counts of lymphocytes, CD3^ + ^T cells, and CD4^ + ^T cells could predict the severity of *C psittaci* pneumonia. Thus, mNGS examination is helpful in early diagnosis, and prompt treatment generally leads to a good prognosis.

## Acknowledgments

The authors thank all the clinicians, microbiologists, and radiologists who have assisted this research.

## Author contributions

**Conceptualization:** Hui Mai, Xiaoying Tan.

**Data curation:** Limin Xu, Hui Mai, Xiaoying Tan, Yubin Du, Changquan Fang.

**Formal analysis:** Limin Xu, Hui Mai, Xiaoying Tan, Yubin Du.

**Investigation:** Xiaoying Tan, Yubin Du.

**Project administration:** Changquan Fang.

**Writing – original draft:** Limin Xu, Ziwen Zhao.
